# Seroprevalence and haemato-biochemical effects of bovine leucosis in buffalo, Punjab, Pakistan

**DOI:** 10.17221/57/2023-VETMED

**Published:** 2023-10-24

**Authors:** Abdul Rahman, Muhammad Kashif, Amar Nasir, Syed Ehtisham-Ul-Haque, Habib Ullah, Arbab Sikandar, Ishtiaq Ahmed, Aziz Ur Rehman, Muhammad Adnan Saeed, Muhammad Waseem Nazar, Muhammad Rizwan, Sidra Saher, Arshad Abbas

**Affiliations:** ^1^Department of Clinical Sciences, University of Veterinary and Animal Sciences, Lahore, Sub-campus, Jhang, Punjab, Pakistan; ^2^Department of Basic Sciences, University of Veterinary and Animal Sciences Lahore, Sub-campus, Jhang, Punjab, Pakistan; ^3^Department of Pathobiology, University of Veterinary and Animal Sciences, Lahore, Sub-campus, Jhang, Punjab, Pakistan; ^4^Department of Clinical Sciences, Bahauddin Zakariya University, Multan, Punjab, Pakistan; ^5^Faculty of Veterinary and Animal Sciences, Gomal University, D.I. Khan, Khyber Pakhtunkhwa, Pakistan

**Keywords:** biochemical parameters, bovine leucosis, ELISA, seroprevalence

## Abstract

Enzootic bovine leucosis is caused by bovine leukaemia virus (BLV), a *Deltaretrovirus* belonging to the family *Retroviridae*. BLV causes huge economic losses to the dairy industry in the form of decreased milk production, premature culling, and poor reproductive performance of the animals. The aim of the present study was to determine the seroprevalence of BLV infection in buffalo in two districts of Punjab, Pakistan. A total of 384 samples were collected and analysed using a commercial indirect enzyme-linked immunosorbent assay (ELISA) to investigate the seroprevalence of BLV through the detection of the anti-BLV gp51 antibody. A predesigned data questionnaire proforma was employed to find out the association of risk factors with disease. Overall, 18.2% of buffaloes were seropositive for BLV in the study population. The results revealed a significant association (*P* < 0.05) of age with BLV infection. Furthermore, milk yield and pregnancy had a significant association with the seroprevalence of BLV infection in buffalo whereas no significant association was found with sex, breeding, and health status. Biochemical and oxidative stress markers revealed a significant decrease in liver enzymes alanine transaminase (ALT) and aspartate transaminase (AST), glutathione peroxidase (GPX), and superoxide dismutase (SOD) in seropositive animals as compared to healthy animals. It is concluded that BLV has a considerable prevalence in buffalo in Punjab, Pakistan and there is a dire need to investigate the disease epidemiology at both national and international levels and strategies should be developed to implement an effective control program.

Livestock is an important sector playing a significant role in the national economy of Pakistan. The total population of buffalo in Pakistan is 42.4 million, yielding a total milk production of 38 363 tons annually. This sector added 60.1% in agriculture value and 11.5% in GDP. More than 80 million rural families are attached to this sector and gain 30–40% of their income from this source (Pakistan Economic Survey 2020–2021; Ministry of Finance 2021).

Bovine leucosis is an infectious, blood-borne, and neoplastic disease naturally occurring in cattle but other species may also be affected. It is also known as bovine lymphosarcoma or bovine leukaemia (Mousavi et al. 2014) and is caused by the bovine leukaemia virus (Selim et al. 2020b). BLV was discovered in 1871 by observing the appearance of yellow nodules on an enlarged spleen (Gillet et al. 2007). The pathogen is a single-stranded diploid RNA virus belonging to the genus *Deltaretrovirus* and family *Retroviridae* (Polat et al. 2015). BLV causes heavy economic losses to the dairy industry in the form of decreased milk production, premature culling, and by drop in the reproductive performance of the animals. Indirectly, this disease causes economic losses by means of bans imposed on the importation of BLV-infected animals from affected regions (Selim et al. 2020a). In the USA the direct losses to the dairy industry due to BLV infection are more than $500 million yearly (Rhodes et al. 2003).

Bovine leucosis has three pathological forms; asymptomatic form, persistent lymphocytosis, and lymphosarcoma. Mostly, animals infected with BLV manifest no clinical signs and remain healthy, about 30% act as carriers, and > 5% exhibit lymphosarcoma (Mousavi et al. 2014). This virus is located in the blood lymphocytes of cattle infected with enzootic bovine leucosis. The horizontal transmission may occur through the blood of infected cattle (i.e., use of contaminated equipment like needles etc.). Mechanically transmission of the BLV may be possible by haematophagous flies. Vertical transmission may result from the ingestion of colostrum or milk from infected cows to calves (Kobayashi et al. 2010).

BLV infection is diagnosed by different methods. There are two methods for the detection of BLV, antibody-based serological tests and proviral genome by PCR detection. Agar gel immunodiffusion (AGID) test and enzyme-linked immunosorbent assay (ELISA) are the most commonly used serological tests for BLV detection. AGID is less sensitive than ELISA, commonly used for routine diagnosis.

Enzootic bovine leucosis is distributed worldwide, and its prevalence differs from region to region. In North and South America, the highest prevalence was reported. In Colombia, Venezuela, Chile, and Uruguay, the individual BLV infection was reported from 30% to 50%. More than 50% was noted in Brazil. According to the International Organization of Epizootics (OIE), BLV infection was reported in Indonesia, Taipei (P.R. China), and Mongolia. In Middle Eastern countries, BLV prevalence is lower as compared to other regions of the world, except Iran and Türkiye where herd level prevalence reaches up to 64.7% and 48.3%, respectively (Rodriguez et al. 2011). In the previous study, we also found a moderately high prevalence of BLV in cattle in our lab in Punjab, Pakistan (Ullah et al. 2023). Keeping in view these data, the present study was designed to find out the seroprevalence of BLV in buffalo along with associated risk factors, and biochemical and haematological variables.

## MATERIAL AND METHODS

### Study area and sample collection

**Figure 1:**
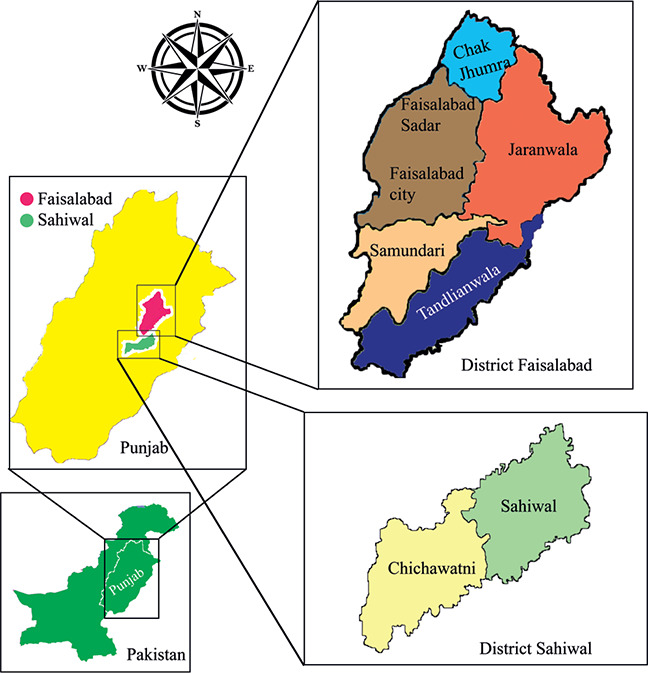
The picture with geographic regions of study and the sites of sample collection

This study was conducted in different areas of Faisalabad and Sahiwal regions, Punjab, Pakistan (Figure 1). The blood samples were randomly collected from buffaloes reared in different villages of the study population. A questionnaire was designed to collect data about different risk factors associated with BLV infection (i.e., age, sex, breed, herd size, herd type, health status, and type of feed). The sample size was 384 with an expected prevalence of 50%. A 3 ml fresh blood was collected aseptically from the jugular vein in two tubes with ethylene diamine tetraacetic acid (EDTA) and gel/clot activator and transported to the laboratory for analysis. The collected blood samples were centrifuged at 2 000* × g* for 10 min to separate serum. The sera were transferred into Eppendorf tubes and stored at –18 °C till further processing for ELISA.

### Serological examination

All the serum samples were examined serologically using an ELISA kit (Anti-BLV gp51 Antibody Detection; IDvet, Grabels, France) to detect the antibodies against BLV according to the manufacturer’s instructions. All the reagents were brought to room temperature (21 °C* ± *5 °C) before use and homogenised by shaking using a vortex. Briefly, 80 μl of dilution buffer was added to each well of the 96-well plate, then 20 μl of positive and negative control were added into duplicate wells, and then 20 μl of each sample was added to the remaining wells. The plates were covered with aluminium foil and incubated for 45 min at 21 °C (± 5 °C).

After incubation, three washings were carried out with 300 μl of wash solution for each well. After washing, 100 μl of conjugate was added into each well and incubated for 30 min at 21 °C (± 5 °C). After washing 100 μl of substrate solution was added to each well of the plate and incubated for 15 min at 21 °C (± 5 °C). Then, 100 μl of stop solution was added to each well. The optical density of colour development was read with the help of an ELISA reader at 450 nm (EL10A ELISA Reader; Biobase, Jinan, P.R. China).

The test was validated when the mean value of the negative control (NC) optical density (OD) was greater than 0.7 whereas the mean value of the optical density of the positive control was less than 30% of the optical density of the negative control. The sample to positive ratios (S/N) was calculated using the formula:

S/N (%)=OD sample/OD NC×100 (1)

When the S/N (%) was equal to or less than 50%, the animal was considered positive for BLV antibodies. When the S/N (%) of the sample was greater than 50% and less than 60%, the sample was doubtful. When S/N (%) was equal to or greater than 60%, the sample was considered negative.

### Biochemical parameters evaluation

Liver enzymes alanine transaminase (ALT) and aspartate aminotransferase (AST) were measured by using an automated chemistry analyser. For the evaluation of ALT and AST, serum samples of seropositive (*n *= 5) and seronegative (*n *= 5) buffaloes were determined. Glutathione peroxidase (GPX) and superoxide dismutase (SOD) levels in seropositive and seronegative buffaloes were measured by using commercially available kits (Ransod; Randox, Crumlin, UK).

### Haematological examination

For haematological examination, fresh blood of seropositive (*n *= 5) and seronegative (*n *= 5) buffaloes were examined.

Haematological variables (total leukocyte count, lymphocytes, monocytes, haemoglobin, mean corpuscular haemoglobin, mean corpuscular haemoglobin concentration, red blood cells count, mean corpuscular volume, packed cell volume, and platelets) were measured by using Exigo, automatic haematology analyser (H400 Boul Medical; Exigo, Spanga, Sweden).

### Statistical analysis

The statistical analysis was performed using SPSS-BRM v26 (IBM, USA). The chi-square test was used to check the seroprevalence of bovine leucosis in buffalo.

Paired sample *t*-test was used for the analysis of biochemical and haematological variables in seropositive and seronegative animals. The effects of age, sex, pregnancy, milk yield, health status, and breeding on infection were evaluated using logistic regression analysis.

The *P*-value (*P* < 0.05) was considered statistically significant.

## RESULTS

Out of 384 samples of buffalo blood, 70 animals were positive for BLV, and the overall prevalence was found 18.2% in Punjab, Pakistan. Buffaloes in the district of Faisalabad presented a higher prevalence of 28.6% as compared to the Sahiwal district of 7.8%.

There was a significant difference (*P *< 0.05) in the prevalence of bovine leucosis between the two districts (Table 1).

**Table 1:** Seroprevalence of bovine leucosis in Buffalo in Faisalabad and Sahiwal districts

Districts	Examined	Positive	Prevalence (%)	Odd ratio	*P*-value
Faisalabad	192	55	28.6	4.73	0.000
Sahiwal	192	15	7.8	–	
				
Overall	384	70	18.2	–	

The age, pregnancy, and health status of the animal were significantly associated with this disease whereas the other factors including sex, breeding, and milk yield presented a non-significant association (*P *> 0.05) with bovine leucosis (Table 2).

**Table 2:** Risk factors associated with bovine leucosis in Faisalabad and Sahiwal districts

Variables	Variable level	Positive/total	Prevalence (%)	Odd ratio	*P*-value
Sex	female	69/374	18.4	3.92	0.495
	male	1/10	10.0		
Age	≥ 2 years	2/54	3.7	0.38	0.000
	≥ 4 years	14/115	12.2		
	≥ 6 years	54/215	25.1		
Pregnancy	pregnant	42/93	45.2	5.66	0.000
	non-pregnant	27/281	9.6		
Breading	AI	38/211	18.0	0.67	0.902
	bull	32/173	18.5		
Milk yield	high	7/81	8.6	0.57	0.042
	medium	10/87	11.5		
	low	52/206	25.2		
Health status	healthy	46/306	15.0	4.95	0.001
	disease history	24/78	30.8		

AI = artificial insemination

The activity of liver enzymes ALT and AST was significantly increased in seropositive buffalo as compared to seronegative buffalo. The activity of the antioxidant enzyme, GPX was markedly lower in BLV-infected animals as compared with seronegative buffalo whereas there was a significant decrease in SOD activity in seropositive buffalo as compared with seronegative animals (Table 3).

**Table 3:** Serum biochemical values of seropositive and seronegative buffaloes (mean** ±** standard deviation, SD)

Serum biochemical parameters	Seropositive animals	Seronegative animals	*P*-value
ALT (μkat/l)	1.329 ± 0.079	0.855 ± 0.102	0.000
AST (μkat/l)	2.889 ± 0.097	2.032 ± 0.031	0.003
GPX (μkat/l)	20.581 ± 0.557	33.259 ± 0.301	0.002
SOD (μkat/l)	0.028 ± 0.029	0.051 5 ± 0.001	0.001

ALT = alanine transaminase; AST = aspartate aminotransferase; GPX = glutathione peroxidase; SOD = superoxide dismutase

The mean values of haematological variables in buffalo are shown in Table 4.

**Table 4:** Haematological variables (mean ± SD) in seronegative and seropositive buffalo

Haematological variables	Seropositive animals (mean ± SD)	Seronegative animals (mean ± SD)	*P*-value
Total leukocyte count (10^3^/μl)	8.48 ± 0.27	7.82 ± 0.53	0.000
Lymphocytes (%)	3.50 ± 0.52	3.18 ± 0.33	0.000
Monocytes (%)	1.63 ± 0.07	0.49 ± 0.89	0.046
Haemoglobin (g/l)	1.11 ± 0.97	1.04 ± 0.08	0.071
Mean corpuscular haemoglobin (pg)	18.54 ± 0.37	18.40 ± 0.42	0.155
Mean corpuscular haemoglobin concentration (g/l)	3.68 ± 0.07	3.55 ± 0.50	0.076
Red blood cells (10^6^/μl)	7.612 ± 1.07	6.24 ± 0.56	0.001
Mean corpuscular volume (fl)	56.84 ± 1.54	54.46 ± 1.52	0.085
Packed cell volume (%)	37.96 ± 4.49	31.10 ± 2.35	0.002
Platelets (10^3^/μl)	771.60± 41.07	741.60 ± 39.67	0.115

fl = femtoliter; pg = pictogram; μ = micro

*P*-values of the variables in the same column are statistically significantly different (*P* < 0.05)

The values of total leukocyte count, lymphocytes, monocytes, red blood cells, and packed cell volumes were significantly higher (*P *< 0.05) in seropositive animals as compared with seronegative animals whereas the values of haemoglobin, mean corpuscular haemoglobin, mean corpuscular haemoglobin concentration and mean corpuscular volume were comparable (*P *> 0.05).

## DISCUSSION

Bovine leucosis is an infectious disease and it causes huge economic losses to the dairy industry. It is an asymptomatic disease affecting the immune system leading to production losses. In a previous study in our lab (Ullah et al. 2023) we found that this disease is highly prevalent (31.3%) in dairy cattle of Punjab, Pakistan and there was a significant association of age, pregnancy, breeding method, milk yield, and health status of seropositive animals with bovine leucosis. The prevalence was higher in animals that were older, pregnant, artificially inseminated, low milk producers, and had a history of ailments but in this study, it has been observed that disease is also prevalent in buffaloes in Pakistan. This is of serious concern that the infection is spreading in regional animals which is an alarming situation for a poor country like Pakistan and neighbouring countries. In the present study, the overall prevalence was 18.2% in Faisalabad and Sahiwal districts. A previous study conducted by Selim et al. (2020a) in Kafr el-Sheikh governorate, Egypt showed that the presence of antibodies against BLV was 9% in buffaloes. Esmailnejad et al. (2020) reported that 52% of buffaloes were positive for BLV in Khuzestan province, Southern Iran. Another study conducted in Pakistan observed 0.8% of buffaloes being positive for anti-BLV antibodies (Meas et al. 2000). The difference in the prevalence may be due to differences in the geographical aspects, population density, housing system, and animal-raising practices.

In the current study, the distribution of BLV prevalence was not significantly different in male and female animals. However, a higher prevalence was noted in females (18.4%) than in males (10%). This indicates that both females and males are prone to BLV infection. The possible reason for the high incidence in females may be due to a variety of stress-inducing physiological processes such as lactation, breeding, oestrus cycles, and pregnancy that females experience in their lives and also due to the importation of unscreened heifers. The incidence of BLV infection was also found to be associated with the age of animals. It was revealed that BLV infection is most prevalent in older animals. The buffaloes older than six years of age had higher seropositivity (25.1%) than at two years of age (3.7%). This high prevalence in older animals may be attributed to a long life span confronting more exposure to BLV infection and making elderly animals more prone to infections. This finding is consistent with several earlier studies with respect to the significant disparity between age groups (Selim et al. 2020b).

In the present study, a significant association was observed between pregnancy and BLV infection in buffaloes. The seropositivity was high in pregnant animals (45.2%) compared to the non-pregnant ones (9.6%). Our results are in accordance with other studies (Ali et al. 2019; Selim et al. 2020b). The high prevalence in pregnant animals may be due to gestation stress affecting the immune system which gives more chance to BLV infection.

The current study showed a non-significant difference in the association of infection with regard to artificial insemination and natural mating. The prevalence of BLV infection through artificial insemination was 18% and natural mating 18.5%; this result indicated that BLV may be equally transmitted through both methods. However, these results are contrary to Ramiz et al. (2021) who found high seropositivity in females after artificial insemination as compared to natural service.

Our results show a significant association of BLV infection with milk production. The prevalence of BLV infection reduced the milk yield. This could be attributed to the deterioration of animal health over time due to decreased feed intake, frailty of infected animals, and weight loss. Similar findings have been also observed in a previous study (Norby et al. 2016). A significant association of BLV infection with animal health history was observed. The seroprevalence was high in those animals possessing a history of previous diseases such as mastitis, repeat breading, abortion, and infectious diseases. This may be ascribed to increased susceptibility to BLV infection as a result of downregulation of immune status due to previous disease.

In terms of liver enzymes, significantly increased activities of ALT and AST were observed in seropositive animals. The increased activity of ALT indicated that BLV virus infects the liver through portal circulation and resides in the hepatocytes and liver parenchyma, resultantly increasing the enzyme concentration in the blood. These findings are in accordance with the study conducted by Ali et al. (2019) who reported similar results. Leukemic lymphocytic cell infiltration in hepatic tissues may cause liver function abnormalities that are manifested in the early stages of leucosis. Another study (Akalin et al. 2015), contradicted our findings that the activity of ALT in seropositive animals remained unaltered. The increased activity of another liver enzyme, AST, in seropositive animals is consistent with Ali et al. (2019) who also found a substantial increase in AST activity in seropositive animals. However, Nikolay et al. (2013) reported a decreased AST activity in seropositive animals.

Oxidative stress is a vibrant concern in the death or transformation of living tissue. In this study, GPX and SOD enzymes were significantly decreased in seropositive animals as compared to healthy animals. Viral infections can alter the oxidative status by increasing the formation of nitric oxide and by inhibiting the enzyme involved in the oxidative stress within the host which may lead to decreased activities of these enzymes. The current findings are in accordance with the studies (Souza et al. 2011; Ali et al. 2019) describing a marked decrease in glutathione peroxidase activity and markers of oxidative stress in BLV-infected animals. The values of total leukocyte count, lymphocytes, monocytes, red blood cells, and packed cell volumes were significantly higher in BLV-infected animals. These may be due to the severity of the viral infection and a shift to the left, in which the excessive demand of differentiation and maturation of the cells occurs after viral infection. The values of haemoglobin, mean corpuscular haemoglobin, mean corpuscular haemoglobin concentration, and mean corpuscular volume were similar because of no issue of malnutrition or any other injury in animals under the study.

It is concluded that BLV infection is present in dairy buffaloes in Sahiwal and Faisalabad regions of Punjab, Pakistan and it affects the biochemical and haematological indicators of the infected animals in the presence of various pathological alterations leading to liver function disorders and physiological disorders in the host. A greater degree of effective measures should be undertaken by government officials and authorities to control this economically important and neglected disease. Farmers rearing buffaloes should be encouraged to carry out the screening of all animals at dairy farms, adopt good husbandry practices, and eliminate the infected animals.
